# Mechanical, Electrical and Rheological Behavior of Ethylene-Vinyl Acetate/Multi-Walled Carbon Nanotube Composites

**DOI:** 10.3390/polym11081300

**Published:** 2019-08-02

**Authors:** Nicoleta-Violeta Stanciu, Felicia Stan, Ionut-Laurentiu Sandu, Florin Susac, Catalin Fetecau, Razvan-Tudor Rosculet

**Affiliations:** Center of Excellence Polymer Processing, Dunarea de Jos University of Galati, 47 Domneasca, Galati 800008, Romania

**Keywords:** carbon nanotubes, nanocomposites, polymer–matrix composites (PMCs), mechanical properties, electrical properties, rheology, injection molding

## Abstract

This paper investigates the rheological, mechanical and electrical properties of a Ethylene-Vinyl Acetate (EVA) polymer filled with 1, 3 and 5 wt.% multi-walled carbon nanotubes (MWCNTs). The melt flow and pressure-volume-Temperature (*pvT*) behaviors of the EVA/MWCNT composites were investigated using a high-pressure capillary rheometer, while the electro-mechanical response was investigated on injection-molded samples. Rheological experiments showed that the melt shear viscosity of the EVA/MWCNT composite is dependent on nanotube loading and, at high shear rates, the viscosity showed temperature-dependent shear thinning behavior with a flow index *n* < 0.35. The specific volume of the EVA/MWCNT composite decreased with increasing pressure and MWCNT wt.%. The transition temperature, corresponding to the *pvT* crystallization, increased linearly with increasing pressure, i.e., about 20 to 30 °C when cooling under pressure. The elastic modulus, tensile strength and stress at break increased with increasing MWCNT wt.%, whereas the strain at break decreased, suggesting the formation of MWCNT secondary agglomerates. The electrical conductivity of the EVA/MWCNT composite increased with increasing MWCNT wt.% and melt temperature, reaching ~10^−2^ S/m for the composite containing 5 wt.% MWCNTs. Using the statistical percolation theory, the percolation threshold was estimated at 0.9 wt.% and the critical exponent at 4.95.

## 1. Introduction

In the past ten years, polymer/carbon nanotube (CNT) composites have been used in various industrial applications which require enhanced properties, especially mechanical and/or electromagnetic properties [1,2,3,4,5,6]. However, the mechanical and physical properties of polymer/CNT composites depend on a number of direct and indirect factors which can decisively impact the final properties of the polymer/CNT composites, such as filler characteristics (single/double/multi-walled, aspect ratio, dispersion, distribution and orientation), polymer type, interface (surface treatment, functionalization, surface energy, etc.) and compounding methods, all of which are well documented in the literature [1,2,3,4,5,6]. As with any new material, researchers have to be realistic in their expectations, since the increase of some properties comes with the trade-off of a potential reduction in other characteristics. For instance, well-dispersed CNTs are ideal for improved mechanical properties, while a continuous phase of loosely-packed CNT agglomerates is ideal for improving the electrical conductivity of the polymer/CNT composites [7].

An important aspect of the production of functional and structural parts from polymer/CNT composites with enhanced physical and mechanical properties relies on the processing conditions. Therefore, it is very important for manufacturers to use the right combination of processing conditions to optimize the nanotube dispersion process. However, despite widespread literature about polymer/CNT composites, only a few articles have focused on the effect of manufacturing processes and their processing parameters on the mechanical and functional properties of this class of materials [7,8,9,10,11,12,13,14,15].

From an industrial perspective, the injection molding process is preferred for the manufacturing of complex plastic parts with high tolerance precision, repeatability, large scale-up, minimal scrap losses and little need to finish parts after molding. Manufacturing polymer/CNT composite parts by injection molding require a good knowledge of the processing parameters, since when these materials are melt-processed, their mechanical properties and/or conductivity can be lost [7,11,14,16].

The properties of the polymer/CNT injection molded parts depend on the degree of nanotube alignment/orientation that is induced during the manufacturing process [7,8,16,17]. Melt and mold temperatures, injection velocity, packing pressure and crystallization rate for semi-crystalline polymers are key factors that affect the dispersion, alignment/orientation and/or aggregation of nanotubes, with a strong influence on the resulting properties of the parts. During the injection molding process, the polymer melt is subjected to changes in flow (shear flow and elongational flow) and heat. Depending on the geometry of the part, the direction and the shape of the flow front also change several times during the injection molding, which influences the network of nanotubes in the final parts [18].

The electrical conductivity of the composite reduces as the shear rate (or injection velocity) increases, due to the orientation and alignment of the nanotubes, which leads to the interruption of nanotube-nanotube contacts (loss of tube-tube contacts) [8,16,17]. However, if the shear rate is reduced and the nanotubes are allowed to disorientate and reagglomerate, the electrical volume conductivity of the composite increases [8,9,11,16,17]. This can be done by keeping the melt in the molten state for a longer time before cooling it down [9,11]. Higher melt temperatures lead to the reorientation or recombination of CNTs due to longer residence time in the molten state; consequently, at higher melt temperatures, higher conductivity is obtained [16]. However, the time necessary for reconstruction depends on the mobility of the polymer chains, which increases with increasing melt temperature [8].

The melt viscosity of polymer/CNT composites is also important in the injection molding process. For example, if the composite melt did not show shear thinning, much higher injection pressure will be needed for filling the mold, and the molding of thin-walled parts will probably not be possible at all. When carbon nanotubes are added into the polymer, the shear thinning is even more important, since the addition of CNTs can increase the melt viscosity of the composite [12,16,18,19,20,21,22]. Therefore, in injection molding, the evolution of the CNT network morphology is mainly dependent on the shear flow conditions.

Ethylene-Vinyl Acetate (EVA) copolymer has been extensively used for the encapsulation of photovoltaic modules due to its outstanding properties, such as high electrical resistivity, low processing and cross-linking temperature, low water absorption, good optical transmission and low resin cost [23,24,25,26,27,28]. On the other hand, in recent years, there has been increasing interest in using EVA filled with CNTs for the development of flexible electrically- and thermally-conductive components, including sensors and actuators with reduced weight and improved mechanical performance [15,29,30,31,32,33,34]. While considering the EVA/MWCNT composites for commercial applications, the understanding of how the processing conditions influence the nanotube networks (dispersion and/or agglomeration), and subsequently the rheological, mechanical and electrical properties, is extremely important.

In this regard, the aim of this study is to gain insight into the properties of EVA/MWCNT composites for the incoming fabrication of smart material components by injection molding. This work notably analyzes EVA filled with different MWCNT loadings (1, 3 and 5 wt.%) manufactured by injection molding, and its electrical and mechanical properties. Since shear rheological properties and *pvT* data are important for the injection molding process, the effect of MWCNT loading on the properties of the EVA/MWCNT composite is also investigated using a capillary rheometer. The challenge is to identify the relation between the processing parameters and the carbon nanotube content in order to tailor the mechanical and electrical properties of EVA/MWCNT composites.

## 2. Materials and Methods

### 2.1. Materials and Sample Manufacturing

The composite used in this study is an ethylene vinyl acetate copolymer (EVA) filled with 1, 3 and 5 wt.% of multi-walled carbon nanotubes (MWCNTs). The EVA/MWCNT composites, supplied by Nanocyl S.A. (Sambreville, Belgium), were prepared by melt mixing the EVA (Alcudia PA-420, Repsol; vinyl acetate (VAc) content of 20 wt.%; melt flow rate of 20 g/10 min at 190 °C/2.16 kg [35]) with Nanocyl NC7000™ thin multi-walled carbon nanotubes [36]. The melt compounding temperature was set to 140 °C and the nanotubes were directly incorporated into the melt.

The EVA/MWCNT pellets were injection-molded into dumbbell-shaped tensile specimens with a thickness of 4 mm (according to ISO 527 Standard) on an Arburg Allrounder injection molding machine (Model 320 C 500–170, Arburg GmbH + Co KG, Lossburg, Germany) based on a three-level design 3^2^ (L_9_), with two factors, i.e., melt temperature (140, 160 and 180 °C) and injection pressure (70, 80 and 90 MPa) (see Table S1 of Supplementary Materials). The injection molding machine was set at maximum pressure; thus, the injection pressure represents the pressure needed to meet the flow rate value. The constant processing parameters used in the injection molding process were the injection flow rate (15 cm^3^/s), mold temperature (30 °C), holding pressure (95% of the injection pressure), holding time (10 s) and cooling time (20 s). After injection molding, all specimens were conditioned in a standard laboratory environment for 24 h before further testing.

### 2.2. Differential Scanning Calorimetry (DSC)

To study the effect of MWCNT wt.% on the thermal characteristics of the EVA/MWCNT composites, DSC measurements were carried out using a DSC 1 differential scanning calorimeter (Mettler Toledo Inc., Columbus, OH, USA). Samples weighing about 8 mg were cut from the as-received EVA/MWCNT pellets. In order to remove the thermal history, samples were first heated from 25 °C to 180 °C at 10 °C/min and held at 180 °C for 5 min, and then cooled down to 30 °C at a cooling rate of 10 °C/min to obtain the non-isothermal crystallization curves. This procedure was repeated in a second run.

### 2.3. Melt Shear Viscosity

Melt shear rheology was used to investigate changes in rheological behavior of EVA/MWCNT composites. The rheological measurements were carried out using a high-pressure capillary rheometer (Rheograph 75, Göttfert GmbH, Buchen, Germany). The melt shear viscosity and shear stress of EVA/MWCNT composites were determined as a function of apparent shear rate, in the range of 100 to 5000 s^−1^. The rheological tests were performed at 120, 140, 160, 180 and 200 °C using three dies with a length-to-diameter ratio of 10/1, 20/1 and 30/1, respectively. Before testing, the pellets were vacuum dried for 3 h at 60 °C in a drying system (Raypa, Terrassa, Spain). The Bagley and Weissenberg-Rabinowitsch corrections were applied to the capillary data in order to determine the true melt shear viscosity curves.

### 2.4. Pressure-Volume-Temperature Measurements

The pressure-volume-temperature (*pvT)* behavior was investigated using a capillary rheometer (Rheograph 75, Göttfert GmbH, Buchen, Germany) equipped with a capillary locking system with a piston of 15 mm in diameter.

Dry pellets were gradually loaded into the preheated barrel at 100 °C and pressurized at 1000 N. After 30 min of conditioning at 100 °C, the melt was extruded until about 18 mm of melt was left in the barrel, and cooled to 50 °C. The specific volume was measured during the heating run using an isothermal mode in order of increasing temperature from 50 to 180 °C, at 5 °C intervals. For each temperature step, the pressure was scanned in increasing order in the range of 10 to 1500 bar. The relaxation time between two temperature intervals was set to 30 min.

### 2.5. Solid Density Measurement

The density of the composites was measured by Archimedes’s principle using a density kit fitted on an analytical balance (AB204-S/FACT, Mettler Toledo Inc., Columbus, OH, USA). The EVA/MWCNT composite was weighted both in air and ethanol, and density was calculated from the two weights as follows:

(1) $$\rho_{EVA/CNT}=\frac{m_{A}}{m_{A}-m_{0}}\left(\rho_{0}-\rho_{L}\right)+\rho_{L},$$

in which $m_{A}$ is the weight of the sample in air, $m_{0}$ is the weight of the sample in the ethanol, $\rho_{L}$ is the density of air (0.0012 g/cm^3^) and $\rho_{0}$ is the density of the ethanol at the measured temperature.

For each nanotube wt.%, at least ten determinations were performed, and the average and standard deviation were reported.

### 2.6. SEM Analysis

To evaluate the dispersion of MWCNTs into the EVA matrix, injection-molded samples were fractured in liquid nitrogen and the fracture surfaces subjected to scanning electron microscopy (Quanta 200, FEI, Hillsboro, OR, USA), operating at 20 kV. Before the SEM analysis, the fracture surfaces were coated with a 5–10 nm Au-Pd layer by sputtering.

### 2.7. Tensile Tests

The mechanical properties of the EVA/MWCNT composites were determined using a universal testing machine (model M350–5AT, Testometric Co. Ltd., Rochdale, UK) at room temperature (23 ± 2 °C). The tensile tests were performed at 5, 50 and 100 mm/min, respectively, to evaluate the strain rate sensitivity. The mechanical properties of the samples were evaluated in terms of the elastic modulus (the slope of the stress-strain curves in the elastic region at low strains), tensile strength (the maximum stress), stress at break and strain at break. To ensure repeatability, at least five specimens were tested for each combination of experimental conditions and the mechanical properties represent the average of five tests.

### 2.8. Measurement of Volume Resistivity

The direct current (DC) volume resistance of EVA/MWCNT composites was measured using a two-point probe method under low voltage. The DC resistance was measured using a voltage source (Model 6221, Keithley Instruments, Inc, Cleveland, OH, USA) in combination with a nanovoltmeter (Model 2182A, Keithley Instruments, Inc, Cleveland, OH, USA), as shown in Figure S1. To minimize errors due to the equivalent resistance of the circuit or other factors that can affect the measured resistance, an active cell was designed and manufactured [37,38]. The circuit being measured was enclosed into a Faraday shield to reduce the environmental influence, as shown in Figure S1.

The DC resistance was measured in the flow direction on the central part of the injection-molded specimens, i.e., 40 × 10 × 4 mm^3^. The electrical conductivity was calculated from the electrical resistance, as shown in Section 1.1 of the Supplementary Materials. Graphite conductive paint was applied on the specimen to increase the contact between the electrode and the sample, as illustrated in Figure S1. In this work, the current was predetermined for each carbon nanotube wt.% to obtain a similar level of voltage, i.e., below 10 V. Three currents were applied, as indicated in Table S2 of the Supplementary Materials. For each combination of testing parameters (wt.% and injection molding parameters), at least five independent specimens were tested.

## 3. Results and Discussion

### 3.1. Crystallization and Melting Behavior of EVA/MWCNT Composites

**Figure 1:**
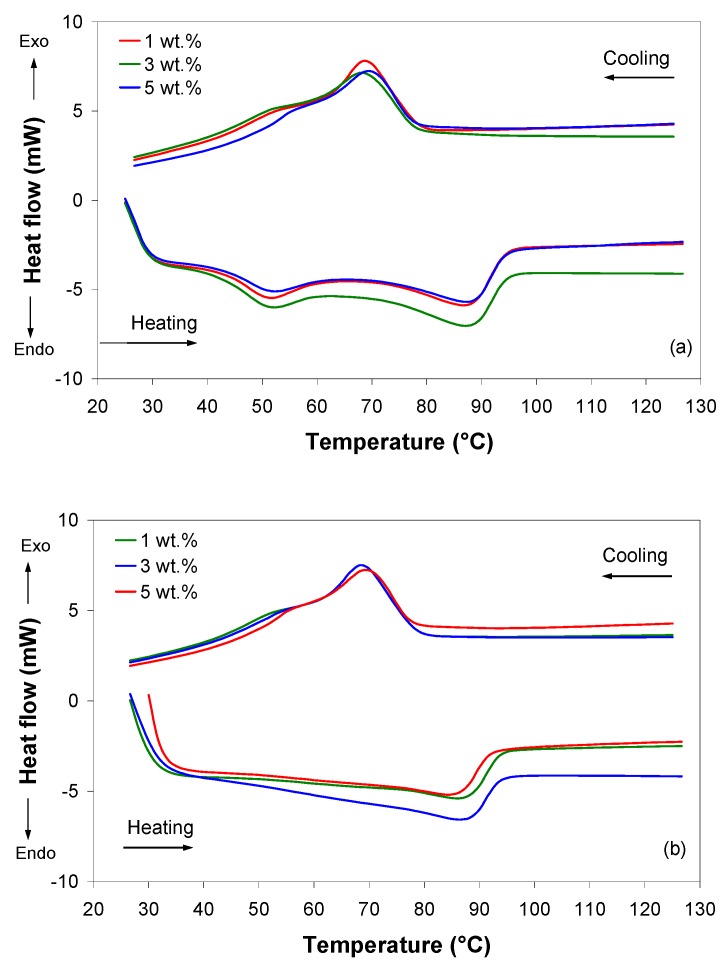
DSC curves at heating and cooling rate of 10 °C/min: (**a**) first DSC scan and (**b**) second DSC scan.

The typical DSC curves for the as-received EVA/MWCNT pellets corresponding to the first heating and cooling scans are shown in Figure 1a. This figure indicates that the first endotherm curve displays two endothermic peaks, while the first exotherm curve displays a single exothermic peak at about 69 °C.

The thermal treatment during the first DSC scan caused a change in the DSC curves for the second heating run, as shown in Figure 1b. For EVA/MWCNT composites heated to 180 °C, cooled and heated once more, the first endothermic peak changed into a shouldered peak and the onset of the melting temperature was difficult to identify because of the broad melting range. The change from a peak into a shouldered peak is an indication of a distribution of crystals with reduced thickness as a consequence of the thermal treatment during the first run, as shown by [25,28]. It is worth noting that for EVA polymers, the width of the melting peak is influenced by the VAc content [25,39]. The width of the melting range increases with increasing VAc content, whereas the melting peak becomes a shouldered peak [25,28,39]. On the other hand, a shouldered peak on the melting curve can indicate more than one component [25,39].

The melting temperature (*T*_m_), crystallization temperature (*T*_c_) and the enthalpy of crystallization (Δ*H*_c_) of the samples, determined from the DSC thermograms, are summarized in Table 1.

DSC results show that the melting and crystallization temperatures of the EVA/MWCNT composite were not influenced by the addition of MWCNT wt.%. On the other hand, the heat released during crystallization was reduced by 16–17 J/g as the nanotube loading increased from 1 to 5 wt.%, which corresponds to a reduction in crystallinity with increasing nanotube loading. In general, at low content, carbon nanotubes act as nucleation sites, promoting polymer crystallization [40]. However, at high contents, carbon nanotube networks provide confinement [39,41] and break the continuity of the polymer matrix; thus, on one hand the crystals cannot grow properly, while on the other, more grain boundary are formed, leading to a reduction in crystallinity. Moreover, it was shown that EVA polymers with high VAc contents have a high amount of VAc defects, and tend to crystallize imperfectly [25,39].

### 3.2. Flow Curves

**Figure 2:**
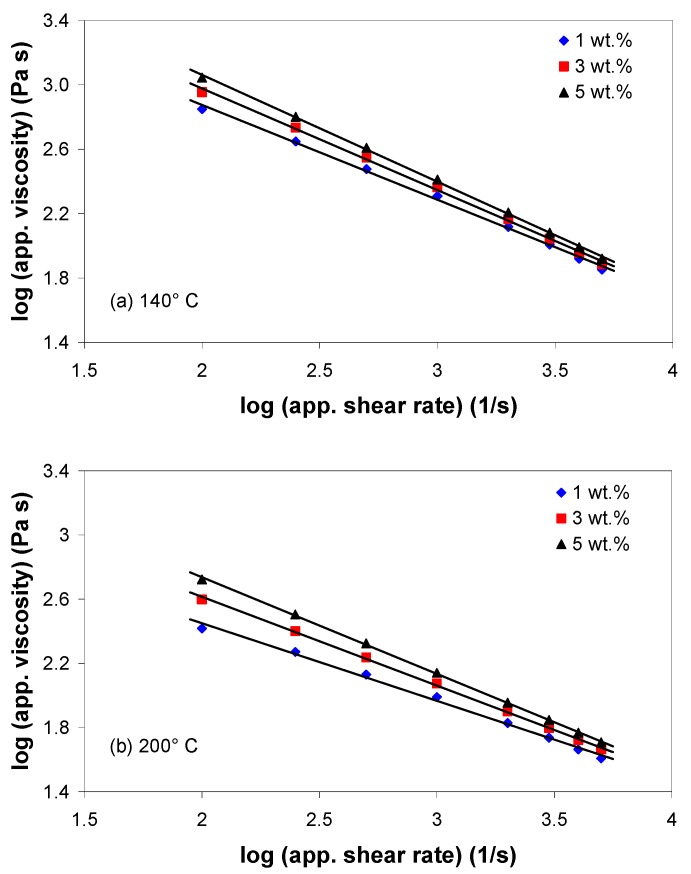
Melt shear viscosity curves at (**a**) 140 °C and (**b**) 200 °C melt temperatures.

Figure 2 presents the variation of the apparent shear viscosity with the apparent shear rate during the capillary flow of EVA/MWCNT composites at 140 and 200 °C, in a bi-logarithmic scale. It can be seen that the apparent shear viscosity decreases linearly with increasing the apparent shear rate, indicating non-Newtonian behavior in nearly the entire range of shear rates. Moreover, the melt shear viscosity of EVA/MWCNT composite increased with increasing MWCNT wt.%, especially at low shear rates. With the increase of nanotube loading, nanotube-nanotube interactions increase, and the polymer chains are generally more restrained. As a result, the viscosity of the composite melt increases.

**Figure 3:**
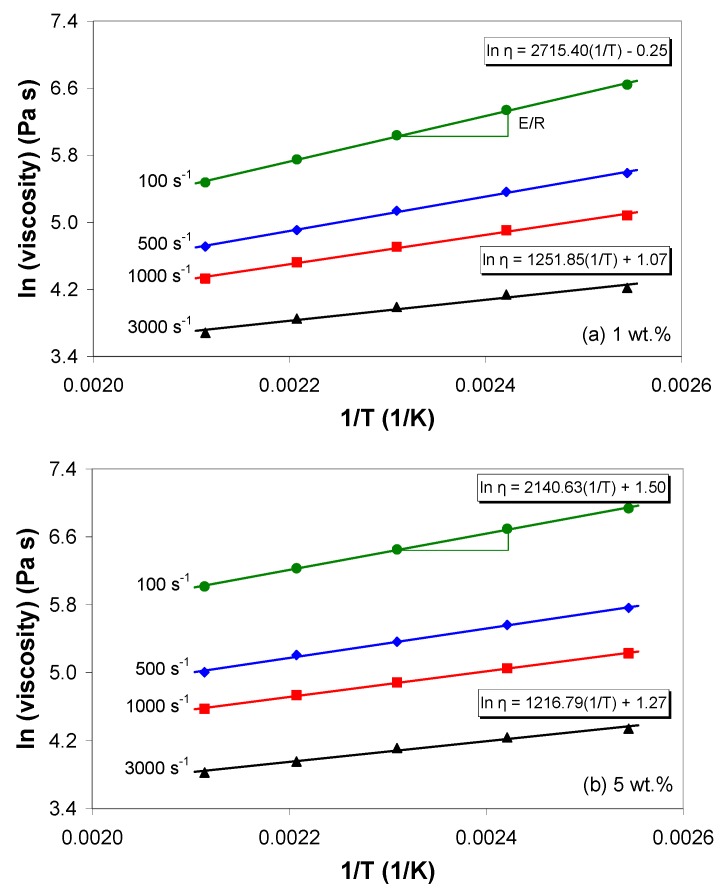
Dependence of melt shear viscosity on temperature and apparent shear rate for (**a**) 1 wt.% and (**b**) 5 wt.% of MWCNTs.

Generally, temperature influences the rheological properties of the matrix, but it can also affect the state of dispersion of the nanocomposites via changes in the particle-particle interactions and in the wettability of the nanotubes with the matrix [42]. The temperature dependence of the melt shear viscosity of the EVA/MWCNT composite is governed by the Arrhenius law, and is depicted in Figure 3 for different apparent shear rates. The slope of the graphs in Figure 3 defines the flow activation energy. Under the experimental conditions used in this work, the flow activation energy decreased with increasing apparent shear rate and MWCNT wt.% (Table 2). Under the same shear rate conditions, the flow activation energy for the composite with 5 wt.% was lower than that of the composite with 1 and 3 wt.%. At a fixed MWCNT wt.%, the more severe the shear conditions, the lower the activation energy.

The decrease of the activation energy with increasing nanotube loadings indicates that the nanotubes are less restricted and have less interaction with the polymer chains, and that more nanotube-nanotube interactions exist [42]. Thus, the temperature dependence of melt shear viscosity should be taken into account when melt-processing these composites. ANOVA analysis of the activation energy shows that both apparent shear rate and nanotube loading have a significant effect on the activation energy (*p*-value < 0.05), with a contribution of about 82% and 17%, respectively.

**Figure 4:**
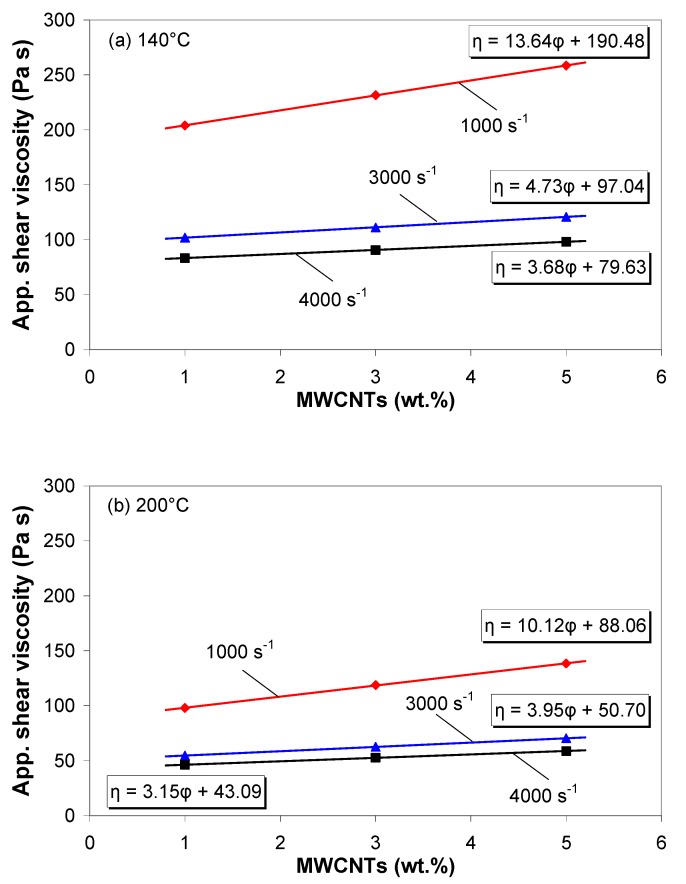
Variation of apparent shear viscosity as a function of MWCNT wt.% at (**a**) 140 °C and (**b**) 200 °C melt temperatures.

Figure 4 shows the variation of the apparent melt shear viscosity as a function of MWCNT wt.% at various apparent shear rates and melt temperatures. As can be seen in Figure 4, the dependence of viscosity on the MWCNT wt.% is to a great degree linear and can be modeled by η = α·φ + β, where φ is the MWCNT wt.% and α and β are two constants [21]. The values of parameters α and β decrease with increasing apparent shear rate and melt temperature, indicating that the sensitivity of the viscosity to the nanotube wt.% is weakened with increasing apparent shear rate and temperature.

**Figure 5:**
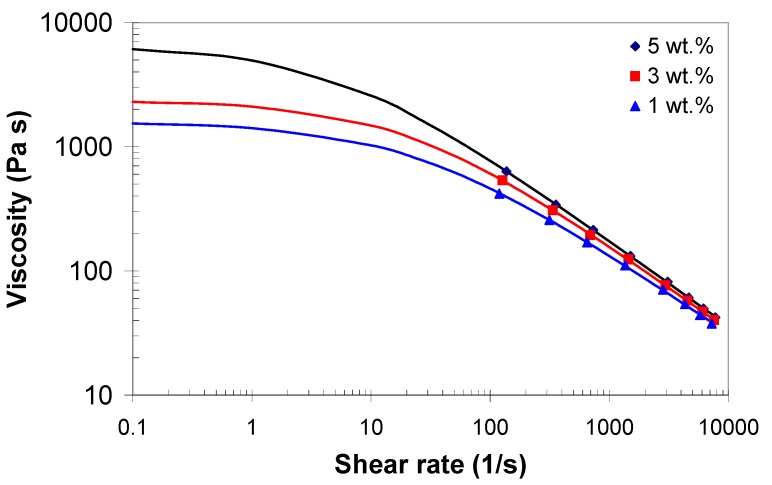
Melt shear viscosity of EVA/MWCNT composite as a function of shear rate at 160 °C (Solid line represents the prediction based on the Cross model).

In Figure 5, the true shear viscosity versus the true shear rate is presented at a 160 °C melt temperature for different MWCNT wt.%. It was found that the melt shear viscosity increases with increasing wt.% and decreases with shear rate, and that all three composites display shear thinning behavior, in agreement with the literature [11,21,43]. The movements of the polymer chains are blocked by the presence of nanotubes increasing the flow resistance of the composite during capillary extrusion, leading to the increase of the shear viscosity of the EVA/MWCNT composites with increasing MWCNT wt.%. However, the extent of the effect of carbon nanotube loading on the melt shear viscosity depends on the degree of shearing. At higher shear rates, the carbon nanotubes and the polymer chains will be fully aligned, and nanotube-nanotube interactions become identical with polymer-polymer interactions [20,21,44,45]. Thus, at high shear rates, the effect of MWCNT loading on the melt shear viscosity is less important.

The decrease in viscosity with increasing shear rates is due to the alignment of nanotubes in the direction of the flow, and to the shear thinning behavior of the composite [8,16]. Also, the shear-thinning behavior can be related to the breaking of interconnected networks between CNTs and/or agglomerates of CNTs [20,21]. This type of rheological behavior can be correlated with the Cross equation [43]. Thus, the experimental data were fitted with a Cross viscosity model

(2) $$\eta =\frac{\eta_{0}}{1+{\left(\frac{\eta_{0}}{\tau *}\dot{\gamma }\right)}^{1-n}},$$

where $\eta_{0}$ is the zero-shear viscosity, τ* is the critical stress level that indicates the transition to the shear-thinning and *n* is the flow-index (shear-thinning index) characterizing the degree of shear-thinning compared to a Newtonian behavior (where n=1).

The Cross model fitted the viscosity data well over the range of experimental data, as shown in Figure 5. The coefficient of determination (*R*^2^) exceeding 0.99 confirms the good applicability of the model to predict the melt shear viscosity. At low nanotube loading (1 wt.%), the Cross model predicts a 3-stage dependence with shear rate: a plateau at low shear rates characteristic of Newtonian behavior, a transition region and a non-Newtonian behavior at high shear rates (>100 s^−1^). On the other hand, at higher nanotube loading, Newtonian behavior disappears and stronger solid-like behavior can be observed, regardless of the shear rates. However, the Newtonian plateau should be treated as apparent, since in the fitting procedure, no experimental values were available for this region.

**Figure 6:**
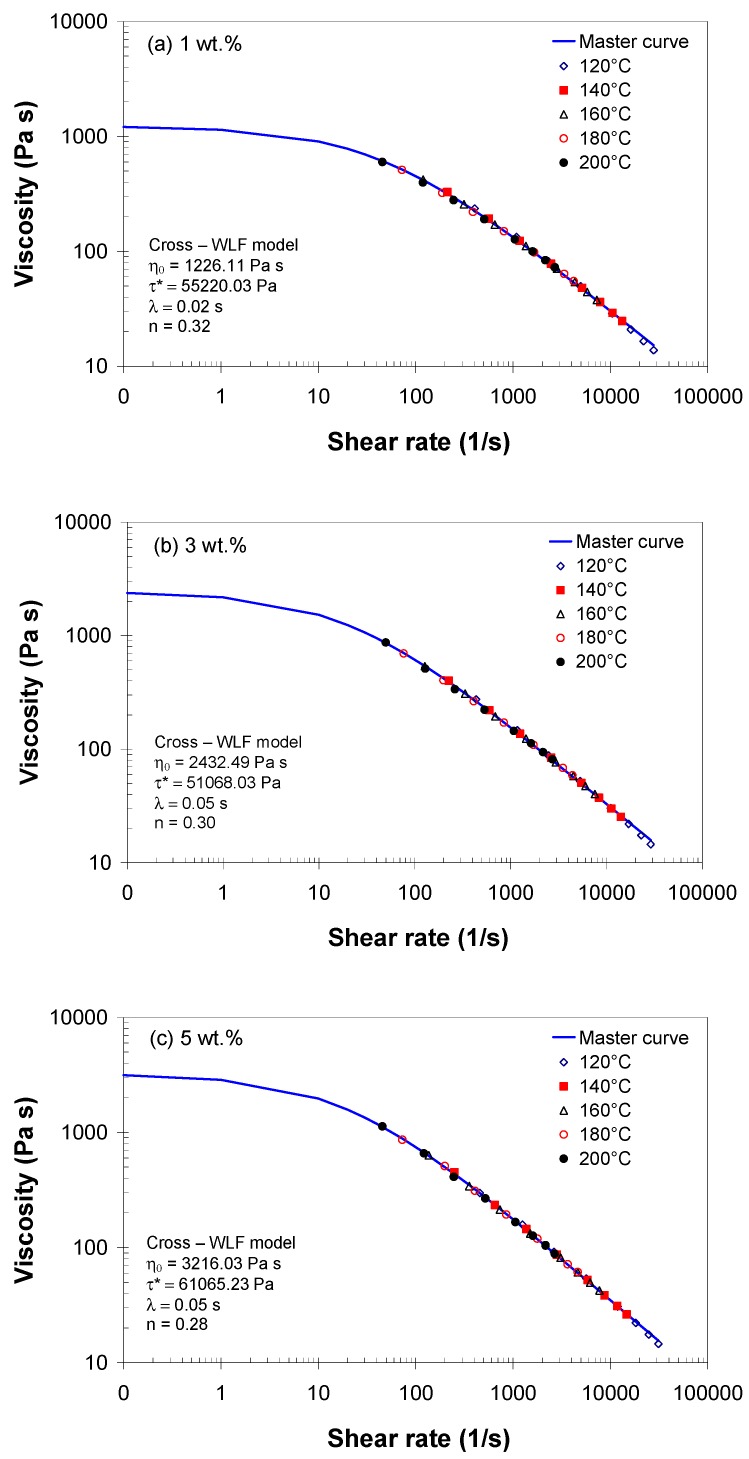
Master curve for EVA/MWCNT composite with (**a**) 1 wt.%, (**b**) 3 wt.% and (**c**) 5 wt.% at a reference temperature of 160 °C.

The time-temperature superposition principle and the WLF equation were used to generate the master shear viscosity curves, which were fitted to the Cross model. The master shear viscosity curves are shown in Figure 6, including the corresponding Cross parameters. The zero-shear viscosity $\eta_{0}$ and the relaxation time $\lambda =\eta_{0}/\tau^{*}$ increased with increasing MWCNT wt.%, while the flow-index *n* decreased with increasing MWCNT wt.%. The higher viscosity of EVA with 5 wt.% can be a disadvantage for the injection molding process of thin or/and complex parts. However, the composite with 5 wt.% exhibits stronger solid-like behavior (*n* = 0.28) as compared with the composite with 1 and 3 wt.%, indicating good processability at higher shear rates.

### 3.3. pvT Data

**Figure 7:**
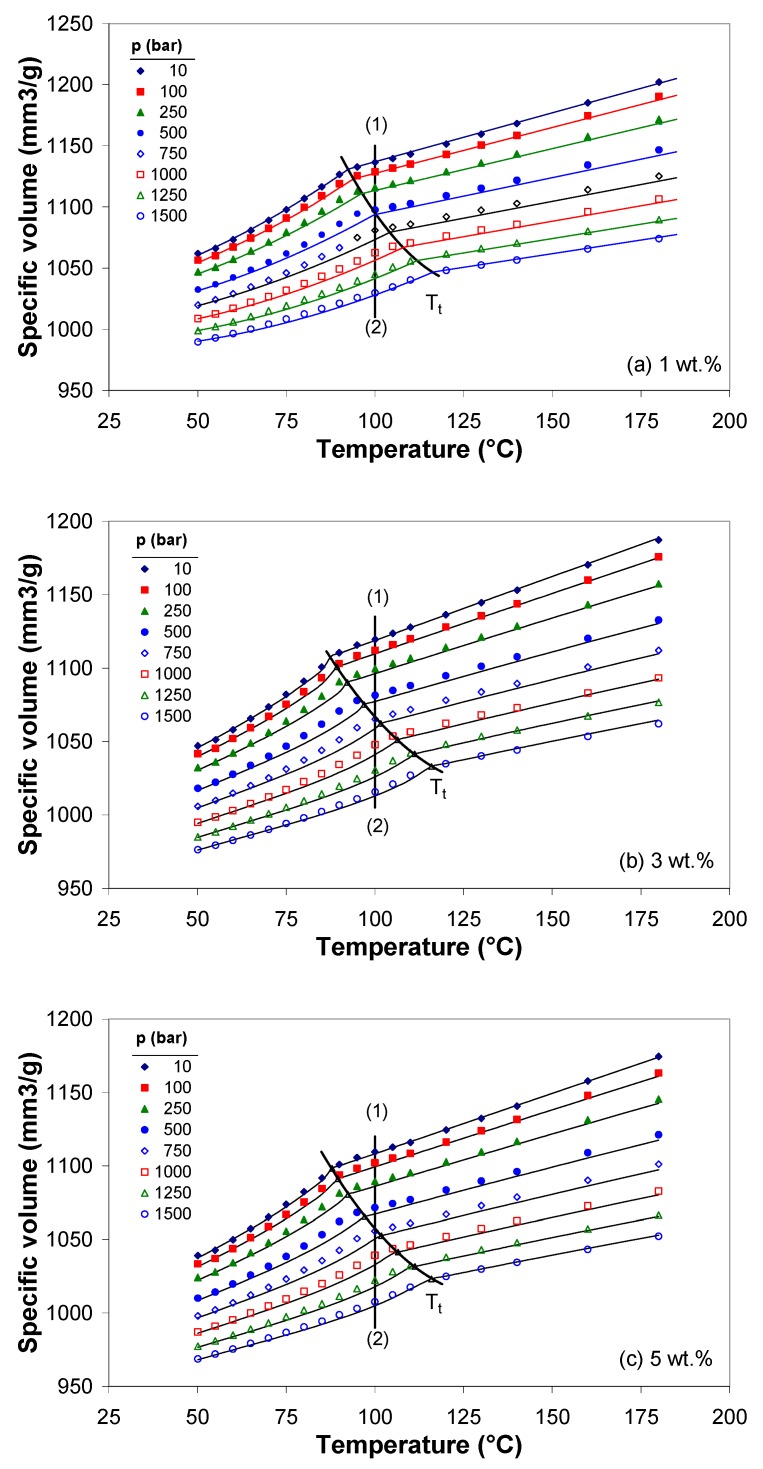
Specific volume as a function of temperature and pressure for EVA/MWCNT composite with (**a**) 1 wt.%, (**b**) 3 wt.% and (**c**) 5 wt.% MWCNTs (The solid line represents the Tait model).

Figure 7 reports the specific volume versus temperature at different pressures for the EVA/MWCNT composite with 1, 3, and 5 wt.%. It is shown in Figure 7 that as the temperature increases, the specific volume passes the solid (or glassy), transition and melt regions, respectively. Moreover, in the melt state, at different pressures, the specific volume of EVA/MWCNT composites changes with temperature linearly, and the transition temperature $T_{t}=T(p)$ shifts toward higher temperatures with increasing pressure.

The solid lines in Figure 7 are calculated from the Tait equation with best fits to the experimental data in the melt and solid states, respectively (The details are given in Section 2.1 of the Supplementary Materials). The Tait fitting parameters for the EVA/MWCNT composite are tabulated in Table 3.

The dependence of transition temperature on the pressure is very important from a practical point of view; it was shown that the service temperature can be increased when the polymers are cooled under pressure [46,47]. As seen in Figure 7, the service temperature of EVA/MWCNT composite can be increased by about 20–30 °C, depending on the nanotube loading. For the EVA composite with 1 wt.%, at 10 bar pressure, the crystallization occurs at around 92.35 °C, while at the highest pressure, crystallization occurs at 116 °C, about 24 °C higher than at atmospheric pressure. For the composite with 3 and 5 wt.% the transition starts around 87 °C at 10 bar, while at 1500 bar, the crystallization starts at 116 °C, 28 °C higher than at 10 bar.

According to Figure 1, the crystallization process takes place in the temperature interval between 70 and 90 °C, which correlates well enough with the *pvT* transition temperature at atmospheric pressure (b_5_).

**Figure 8:**
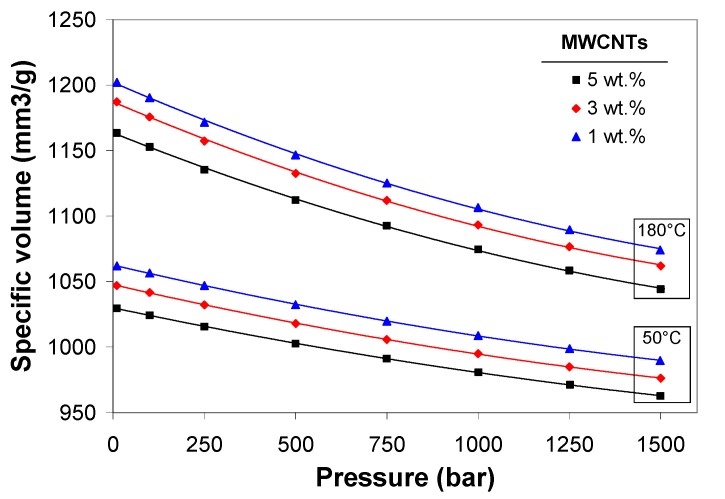
Effect of MWCNT wt.% and temperature on the specific volume in melt and solid state.

Figure 8 illustrates the effect of pressure and MWCNT wt.% on the specific volume at the temperatures of 50 and 180 °C. The change in specific volume between 1 and 5 wt.% is about 2–3%. The decrease of specific volume with increasing nanotube wt.%, indicates that nanotubes have implications on the dimensional stability of the composite and reduce shrinkage. This could be due to the fact that nanotubes are not subjected to shrinkage of compressibility, and therefore, limit the composite shrinkage accordingly.

### 3.4. Morphology of EVA/MWCNT Composite

**Figure 9:**
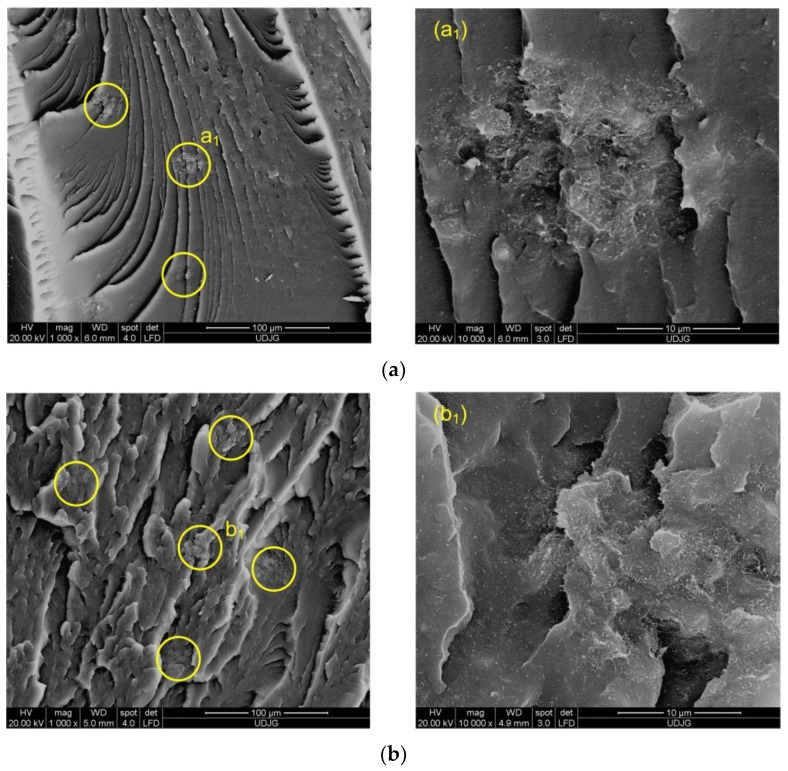
SEM micrographs for fractured surface of the EVA/MWCNT composite with (**a**) 1 wt.% and (**b**) 5 wt.% of MWCNTs (The yellow circles are spherical-like agglomerates).

Figure 9a,b show the SEM images of the injection-molded EVA/MWCNT composite with 1 and 5 wt.%, respectively. The fractured surface of the composite shows many irregularities and, depending on the nanotube loading and injection molding conditions, three different types of distributions could be identified based on the SEM micrographs: single nanotubes, uniformly dispersed nanotubes and spherical like-agglomerates. As shown in Figure 9b, the number of spherical like-agglomerates increased with increasing nanotube loading. The spherical-like agglomerates generally act as stress concentration points [48] and contribute to the poor tensile strength and elongation of the composite.

### 3.5. Mechanical Properties of EVA/MWCNT Composites

**Figure 10:**
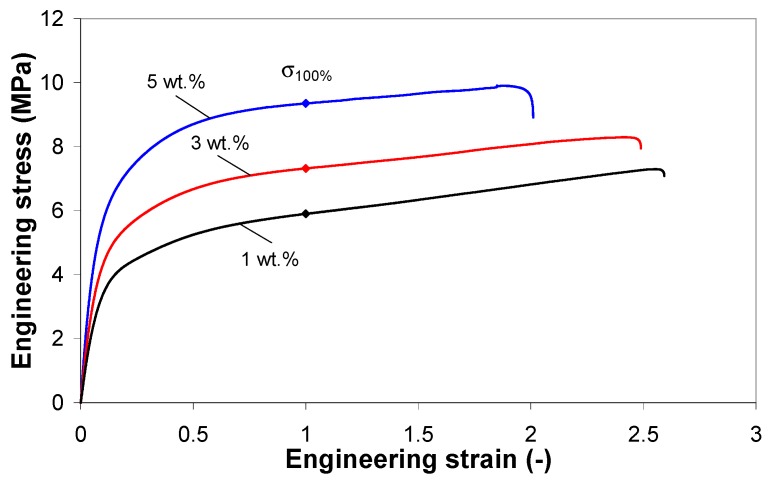
Stress-strain curves for EVA/MWCNT composites at 50 mm/min.

Figure 10 shows the representative stress-strain curves for EVA/MWCNT composites at 50 mm/min crosshead speed. As shown, the EVA/MWCNT composite exhibits a classical mechanical behavior under tension. In addition, the tensile properties of EVA/MWCNT composites change with carbon nanotube content. For instance, the tensile strength at 100% elongation of the composite significantly increases with increasing nanotube content, indicating an effective matrix-to-nanotube transfer.

**Figure 11:**
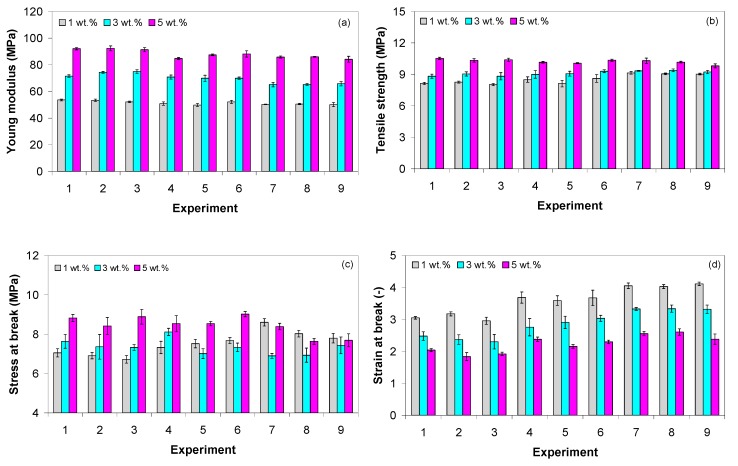
Mechanical properties of EVA/MWCNT composite: (**a**) Young modulus, (**b**) tensile strength, (**c**) stress at break, and (**d**) strain at break at 50 mm/min.

The influence of processing parameters, including the crosshead speed, on the mechanical properties (i.e., elastic modulus, tensile strength, stress at break and strain at break) of EVA filled with MWCNTs is shown in Figure 11a–d. Overall, the mechanical properties of EVA/MWCNT composites increased with increasing nanotube content except for strain at break, which decreases with increasing nanotube wt.%.

The elastic modulus of EVA composites with 5 wt.% MWCNTs increased by 70% when the nanotubes wt.% increased from 1 to 5 wt.%, respectively (Figure 11a). A similar tendency was observed for the tensile strength (Figure 11b), but the reinforcement effect on the tensile strength was lower than expected. The $\sigma_{100\%}$ EVA/MWCNT composite with 3 and 5 wt.% increased by about 25% and 60%, respectively, compared to the value of the composite with 1 wt.%.

The variation of the stress at break of the EVA/MWCNT composite as a function of MWCNT wt.% and crosshead speed is shown in Figure 11c. The stress at break of EVA with 5 wt.% increased on average by 17% when the nanotube content increased from 1 to 5 wt.%, respectively. However, the EVA/MWCNT composites have 20–25 % lower stress at break compared to the EVA matrix (11 MPa [35]), depending on the MWCNT wt.% and processing conditions.

The strain at break decreased with increasing MWCNT wt.%, as shown in Figure 11d. This is the usual consequence of the addition of nanotubes into the polymer matrix. The strain at break dropped to more than 50% for the EVA/MWCNT composite with 5 wt.% compared to the value of the composite with 1 wt.%. On the other hand, strain at break was reduced from about 780% in EVA matrix [35] to about 350% with the incorporation of 1 wt.% of MWCNTs.

**Figure 12:**
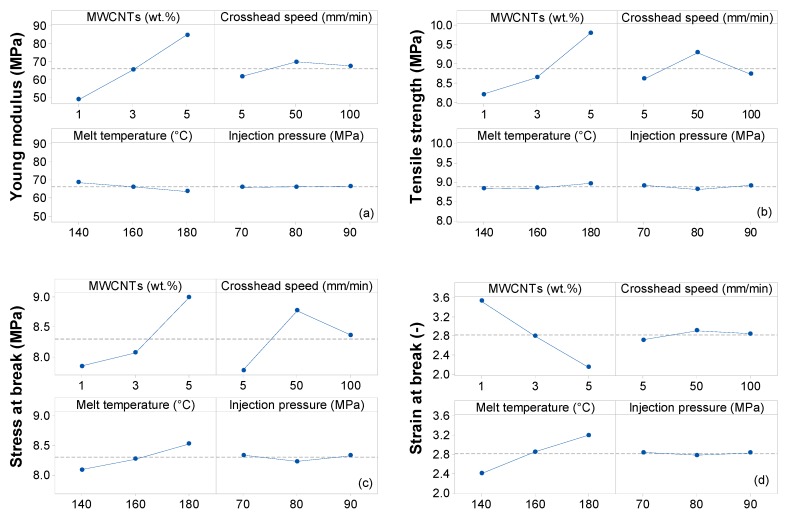
Main effect plots for (**a**) Young modulus, (**b**) tensile strength, (**c**) stress at break, and (**d**) strain at break of EVA/MWCNT composite.

To systematically investigate the influence of each process factor (MWCNT wt.%, crosshead speed, melt temperature and injection pressure) on the mechanical properties of EVA/MWCNT composites, an ANOVA analysis was run for each mechanical property, and the main effect plots were constructed, as shown in Figure 12a–d. The ANOVA analysis, at a typical confidence level of 5%, was implemented on the four factors and the corresponding two-way interactions; the results are reported in Tables S3–S6 of the Supplementary Materials.

For the elastic modulus, based on ANOVA Table S3, MWCNT wt.%, crosshead speed, melt temperature, the interaction of MWCNTs with melt temperature and the interaction of MWCNTs with crosshead speed are significant because their *p*-values are less than 0.05. Injection pressure and any other interactions are not significant, since their *p*-values are higher than 0.05. The main effects plot in Figure 12a confirms these results. Elastic modulus increases with increasing MWCNT wt.%, implying that the external loads are transferred to the nanotubes in terms of shear stress through nanotubes-EVA polymer interface. Moreover, for EVA/MWCNT composites, a higher elastic modulus is obtained at low temperatures. Therefore, high melt viscosity with a rather moderate shear is needed for good dispersion and for the improvement of the mechanical properties of the final parts.

For tensile strength, based on ANOVA Table S4, it was found that the MWCNT wt.%, crosshead speed, the interaction of MWCNTs with melt temperature, the interaction of MWCNTs with crosshead speed and the interaction of crosshead speed with melt temperature are significant (*p*-values < 0.05). Melt temperature and injection pressure show the same pattern, in which the tensile strength remains constant as these two variables increase. In addition, the main effect plot in Figure 12b suggests that tensile strength increases with increasing crosshead speed, but eventually decreases beyond 50 mm/min.

For stress at break, ANOVA Table S5 suggests that the MWCNT wt.%, crosshead speed, melt temperature, the interaction of MWCNTs with both melt temperature and crosshead speed and the interaction of crosshead speed with melt temperature are significant (*p*-values < 0.05). The MWCNT wt.%, melt temperature and crosshead speed show the same pattern in which stress at break increases as the nanotube loading, melt temperature and crosshead speed increases, but decreases beyond 50 mm/min, as illustrated in Figure 12c. Injection pressure did not have a significant influence on the stress at break. The increase of stress at break could be related to the orientation of carbon nanotubes, the alignment of polymer chains in the direction of the flow (which is the same as the direction of the applied force) and good adhesion between the nanotubes and EVA matrix. The decrease of tensile strength with the increase of crosshead speed could be related to the faster occurrence of the loss of interfacial interactions with the matrix.

The *p*-values column in ANOVA Table S6 indicates that the effect of MWCNT wt.%, crosshead speed, melt temperature, the interaction of MWCNTs with melt temperature, the interaction of MWCNTs with crosshead speed and the interaction of crosshead speed with melt temperature on the strain at break is significant (*p*-values are less than 0.05).

A higher melt temperature provides higher strain at break; this effect is more obvious for the composite with low carbon nanotube content, as shown in Figure 12d. On the other hand, the strain at break decreases with increasing MWCNT wt.%. The injection pressure and crosshead speed do not appear to affect the strain at break. A higher melt temperature promotes nanotube migration and reorganization/reagglomeration due to decreased viscosity. Thus, higher melt temperature facilitates the mobility of carbon nanotubes and the formation of higher MWCNT agglomerations, i.e., macro-size particles, which increase the brittleness of the EVA composite and changes the effective volume fraction.

The combination of lower melt temperature (140 °C) and pressure (70 MPa) can successfully increase the elastic modulus, while higher melt temperature (180 °C) and lower pressure (70 MPa) can successfully increase the tensile strength, stress at break and strain at break.

### 3.6. Prediction of Elastic Modulus and Solid Density

Table 4 presents the elastic modulus of the EVA/MWCNT composite predicted by the Halpin-Tsai model with a non-linear shape factor, as shown in Equations (S13)–(S15) of the Supplementary Materials, for a crosshead speed of 100 mm/min. The corresponding experimental values are also presented in Table 4.

The nanotube volume fractions were calculated (0.51%, 1.55% and 2.60%) based on the carbon nanotube and EVA densities of 1.85 g/cm^3^ [36] and 0.940 g/cm^3^ [35], respectively. The average length of the NC7000™ is approximately 1.5 µm while the average diameter is 9.5 nm [36]. Therefore, an average aspect ratio, l/d, of about 150 is expected. As can be seen in Table 4, good agreement is obtained between the experimental and the theoretical results by assuming an exponential shape factor ($\varsigma =(2l/d)\;e^{-0.92\;V_{MWCNT}-1.05}$) for randomly-aligned fillers.

The theoretical density of EVA/MWCNT composites was calculated by the volume-based rule of mixtures assuming densities of 0.940 g/cm^3^ and of 1.85 g/cm^3^ for EVA and MWCNTs, respectively (see Table 4). The conventional rule-of-mixtures provides a good prediction for the density of EVA/MWCNT composites, with the relative error (between the experimental and the predicted values) being less than 1%, as illustrated in Table 4. Regarding the effect of MWCNTs on density, it was observed that the density of the composite slightly increases with increasing MWCNT wt.% (less than 5%).

### 3.7. Electrical Conductivity of the EVA/MWCNT Composite

**Figure 13:**
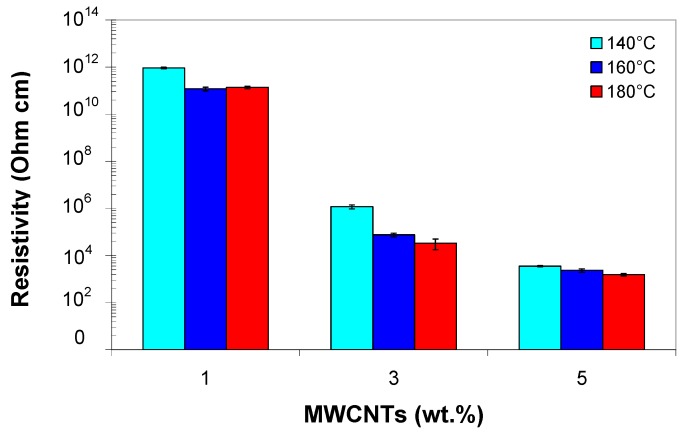
Electrical resistivity of EVA/MWCNT composite as a function of melt temperatures and MWCNT wt.% at 70 MPa injection pressure.

Figure 13 shows the variation of volume resistivity of the EVA/MWCNT composite as a function of carbon nanotube wt.%, for different melt temperatures and 70 MPa injection molding pressure. The error bars in this figure indicate the standard deviation. The volume resistivity of the EVA composite decreases with increasing MWCNT wt.% and melt temperature, as shown in Figure 13. Keeping the injection pressure constant, the resistivity varies within one order of magnitude as the melt temperature increases from 140 to 180 °C; this tendency is almost equal for 1, 3 and 5 wt.%. This could be explained by the fact that once the skin layer is frozen, it can no longer be influenced by a higher melt temperature, and thus, the possibility of nanotubes to reorganize and reagglomerate can only occur in the core region [10]. However, the effect of melt temperature on resistivity is less significant if compared to the effect of nanotube wt.%.

Electrical conductivity in EVA/MWCNT composites, i.e., the inverse of the electrical resistivity, ranges from 10^−10^ to 10^−2^ S/m, depending on the MWCNT wt.%. For the composite with 1 wt.%, the electrical conductivity is about 2 × 10^−10^ S/m, which is two orders of magnitude higher than that of the EVA polymer (typically ranging from 10^−12^–10^−14^ S/m [35]). This indicates that the nanotubes are isolated from each other by the insulating matrix, and that the distance between them is beyond the critical inter-tube distance [49]. As a result of the long distance between carbon nanotubes and the high resistance of the matrix, none of the carbon nanotubes or clusters forms a percolation network, and the potential barrier, due to the interfaces, is too high for electron hopping, as shown by several authors [5,16,50].

The conductivity increased with increasing nanotube loading from 1 to 3 wt.%, indicating the formation of an electrically-conductive nanotube path through which electrons travel along the polymer matrix. As the filler loading approaches 3 wt.%, the electrical conductivity increases by six orders (~10^−4^ S/m) of magnitude as compared with the composite with 1 wt.% (from ~10^−10^ to ~10^−4^ S/m). The inter-tube distance is equal or less than the tunneling distance, but the nanotubes are not in contact with one another [50]. For higher nanotube concentrations (i.e., 5 wt.%), due to the formation of multi-percolating networks through the injection-molded samples, the electrical conductivity increases three orders of magnitude reaching about 10^−2^ S/m. It should be noted that Sabet et al. (2016) [34] reported a conductivity of about 10^−4^ S/m for molded EVA/CNT samples.

**Figure 14:**
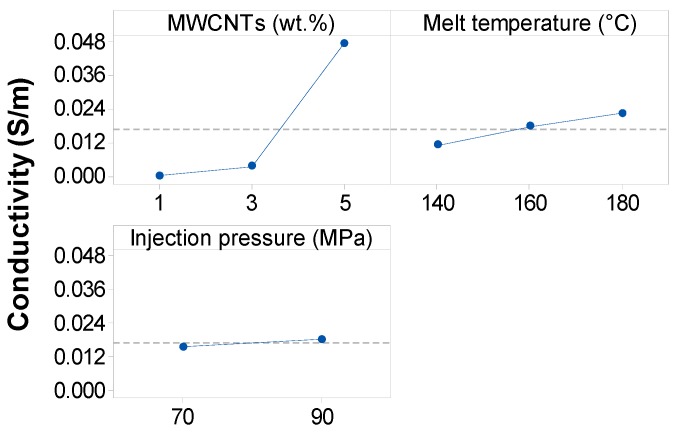
Main effect plots for electrical conductivity of EVA/MWCNT composite.

To facilitate the comparison between different sets of experiments, the data were statistically analyzed using the ANOVA method at the typical confidence level of 5%. Based on ANOVA Table S7, it was found that the MWCNT wt.% (*p* = 0.0), melt temperature (*p* = 0.037) and interaction of MWCNTs with melt temperature (*p* = 0.038) have a significant effect on the electrical conductivity (*p*-values are less than 0.05). However, injection pressure (*p* = 0.301), the interaction of MWCNTs with injection pressure (*p* = 0.717) and the interaction of melt temperature with injection pressure (*p* = 0.57) show no significant influence on the electrical conductivity at 5% significance level, as shown in Table S7. Examining the main effect plots in Figure 14 confirms these results. The increase of EVA/MWCNT electrical conductivity with increasing temperature could be related to the reduction of viscosity. The effect of melt temperature is more important at higher MWCNT wt.%, since higher melt temperature promotes migration, reorientation and reagglomeration of the nanotubes in the molten polymer due to decreased viscosity [11,17]. However, the time necessary for the reconstruction of the MWCNT network depends on the mobility of the polymer chains, which increases with increasing melt temperature but decreases with increasing nanotube wt.% [8,16].

In fact, the conductivity of the EVA/MWCNT composite must be analyzed with respect to processing history, i.e., double processing-extrusion and injection molding, as well as to the orientation of carbon nanotubes during the injection molding process [8,11,16]. It was reported that a slight orientation of the nanotubes increases conductivity, while strong orientation reduces conductivity due to the reduced percolation density [8,11,51].

During the injection molding process, the EVA/MWCNT pellets are screwed into the heated barrel and, thus, subjected to both elongation flow and shear, which can both break up the primary nanotube agglomerates and generate secondary agglomerations. At the gate, depending on the gate size and geometry, the shear rate can be as high as 10^5^ s^−1^; the EVA/MWCNT melt flows through an abrupt contraction and the flow streamlines converge causing acceleration of fluid particle and thus, in addition to shear, also extensional deformation [43]. These severe conditions are expected to break down the initial (primary) carbon nanotube agglomerates which were formed during the dispersion of carbon nanotubes into the EVA matrix, i.e., melt blending in a twin screw extruder. On the other hand, the flow in the mold is non-isothermal, and when the molten polymer flows from a smaller to a larger cross section, it relaxes. In the skin layer, the nanotubes are more oriented due to high shear rates, and when the melt with the oriented CNTs come into contact with the cold mold, the orientation is frozen. In the core, which stays molten for a longer time, the nanotubes remain less oriented (un-orientated) and, possibly, can reagglomerate, forming secondary agglomerates. Once the mold is volumetrically filled, the pressure-controlled packing phase starts to compensate for the shrinking of the part; unlike the filing phase, the packing phase is characterized by flow at low temperature and low shear rate, before melt solidifies; thus, shear flow can both generate and break up secondary agglomerates [8,11,16]. As discussed by Alig et al. (2012) [16], the secondary agglomerates that form during the injection molding process are more loosely packed than the initial agglomerates, and they can be easily destroyed by shear treatment. The formation of agglomerates in the composite might also relate to the growth of the crystalline phase, which expels the nanotubes towards the amorphous phase. The presence of the nanotubes provided a tremendous number of sites for heterogeneous nucleating and, as a result, crystallization onset and peak temperature increased [40,41].

### 3.8. Modeling of Electrical Conductivity

First, the regression analysis was performed in order to establish a direct relation between the electrical conductivity and the processing factors with the highest impact (melt temperature and nanotube wt.%). This model can be written as

(3) $$\begin{array}{cc} \sigma \;(S/m)= & 3.344\times 10^{-2}-3.254\times 10^{-4}\times T_{melt}\;({}^{\circ}C)-2.075\times 10^{-2}\times \phi_{MWCNT}(\mathrm{wt}.\%)+\\ & +2.036\times 10^{-4}\times T_{melt}\;({}^{\circ}C)\times \phi_{MWCNT}(\mathrm{wt}.\%). \end{array}$$

Secondly, the experimental data were fitted to the power-law model. In order to fit the experimental data to the power-law model as in Equation (S16) of the Supplementary Materials, the conductivity values for the optimum injection molding parameters were considered. For electrical conductivity, MWCNT wt.% has the greatest influence, followed by melt temperature and injection pressure. However, the effect of injection pressure is not significant within the analyzed range.

**Figure 15:**
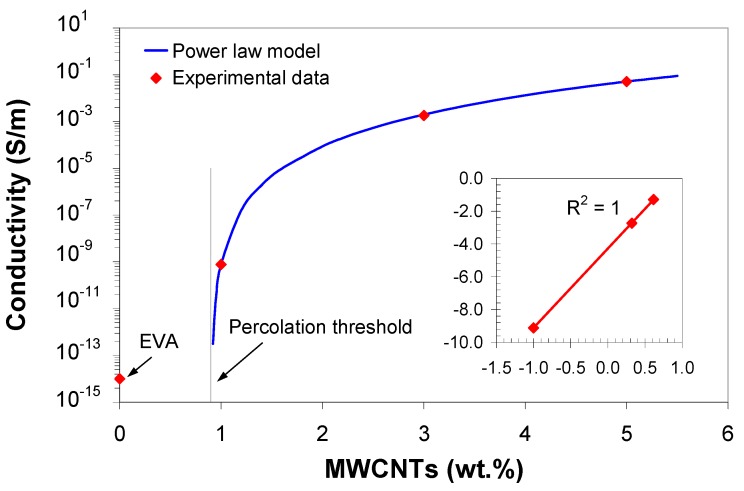
Variation of electrical conductivity as a function of MWCNT wt.% (Experimental data correspond to the optimum injection molding parameters).

Figure 15 shows the experimental conductivity at 180 °C melt temperature and 70 MPa injection pressure versus MWCNT wt.% together with the fitting curve (solid line) given by:

(4) $$\sigma_{DC}\;(S/m)=6\times 10^{-5}\times {\left[\phi_{MWCNT}(wt.\%)-0.9\right]}^{4.95}.$$

The log-log plots of σ versus ($\phi -\phi_{0}$) shown in the inset of Figure 15 reveal linear relationships indicating a good fit (*R*^2^ = 1). Fitting the experimental conductivity to Equation (S16) yields a percolation threshold of 0.9 wt.% and a critical exponent t=4.95.

The predicted $\sigma_{MWCNT}$ is much lower than the corresponding conductivity of NC7000™ nanotubes (~10^6^ S/m [36]). This difference might be attributed to contact resistance between carbon nanotubes or clusters in the composite, which decreases the effective conductivity of the composites [5]. Carbon nanotubes are coated with an insulating polymer layer which results in poor electrical contact between the ends of the carbon nanotubes. Although the power-law model gave good agreement between the theoretical and the experimental data, the choice of the parameters involves some arbitrariness. For example, the critical exponent is much higher than the theoretical value for 3D percolation networks [5,52]. Such a large exponent may be related to the increasing tunneling barriers between the nanotubes, suggesting a broad distribution of the tunneling resistance, and hence, a broad distribution of the inter-particle distance [52].

The polymer usually forms an insulating layer around each nanotube or a gap between two conductive networks, thus intra- and inter- clusters tunneling and hopping take place. Above the threshold, the intra-cluster and agglomerate tunneling mechanism dominates. The power-law model, based on the percolation theory, assumes an ideal system with uniform distribution of the carbon nanotubes [50,53]. In fact, the conductivity of composites containing nanotubes is strongly dependent on the attributes of nanotubes, namely the aspect ratio (l/d) [5], dispersion and orientation of nanotubes within the polymer matrix. However, the distribution of nanotubes is far from being ideal, since during the injection molding process, the MWCNTs are dispersed in the form of agglomerates and orientated along the flow direction.

## 4. Conclusions

The key objective of this work was to determine a set of reliable properties for EVA/MWCNT composites that could be used in design and process development by designers and manufacturers. To reach this goal, we investigated, by different characterization methods, the rheological, electrical and mechanical behavior of an EVA/MWCNT composite filled with 1, 3 and 5 wt.%. The melt shear behavior of the EVA/MWCNT composites was investigated as a function of temperature (120 to 200 °C) and shear rate (75 to 5000 s^−1^). Based on the shear viscosity data, master viscosity curves were constructed using the TTS principle and the Cross model. The *pvT* behavior was also investigated using a high-pressure capillary rheometer and the data fitted to the Tait equation in both liquid and solid states. The electrical and mechanical properties of the EVA/MWCNT composites were investigated on injection-molded samples manufactured at different processing parameters. The results showed that:

- The melt shear viscosity of the EVA/MWCNT composite increases with increasing MWCNT wt.%, especially at low shear rates, and decreases with increasing shear rate and/or temperature. At high shear rates, the EVA/MWCNT composite displays shear thinning (*n* < 0.35), and stronger solid-like behavior is observed at higher nanotube loading.
- The presence of the nanotubes lowers the mobility of the polymer chains and increases the flow activation energy. Evaluation of the melt shear viscosity of the EVA/MWCNT composites gives an Arrhenius flow activation energy of about 12.5 to 22.5 kJ/K mol.
- The specific volume of the EVA/MWCNT composite was found to increase with increasing temperature and decrease with increasing pressure and/or nanotube content. The *pvT* data were well described by the Tait equation both in liquid and solid state.
- The DSC melting and crystallization temperatures are not affected by the presence of the nanotubes, while the enthalpy of crystallization decreases with increasing MWCNT wt.%. However, the *pvT* transition temperature increases linearly with increasing pressure, e.g., the transition temperature increased by 20 to 30 °C as the pressure increased from 10 to 1500 bar, depending on the nanotube loading.
- The mechanical properties of EVA/MWCNT composites were found to be significantly affected by the addition of nanotubes. Incorporation of 5 wt.% MWCNTs into the EVA polymer results in an increase in elastic modulus and yield strength by, respectively, 70% and 20% compared to the composite with 1 wt.% of MWCNTs. On the other hand, the decrease in the strain at break with addition of MWCNTs suggests the formation of MWCNT agglomerates.
- The electrical conductivity of the EVA/MWCNT composite increased with increasing MWCNT wt.% and melt temperature. For 1 wt.% MWCNTs, the electrical conductivity reached about 10^−10^ S/m, whereas the value in the composite with 5 wt.% increased by 6 to 8 orders of magnitude and saturated to a value of 10^−2^ S/m, regardless of the conditions under which they were processed. The electrical conductivity of the EVA/MWCNT composite for optimum conditions (that maximize the conductivity) was found to follow percolation behavior with a threshold mass fraction of 0.9 and higher value of critical exponent 4.95.
- The results recorded for various processing parameters and the ANOVA analysis indicate that, in the investigated range, melt temperature plays a more important role on the mechanical and electrical properties than injection pressure. By controlling melt temperature and MWCNT wt.%, the mechanical and electrical properties can be tailored.

## Figures and Tables

**Table 1:** DSC results for EVA/MWCNT composites.

MWNCTs (wt.%)	1^st^ DSC scan				2^nd^ DSC scan		
	$1^{\mathrm{st}}\;T_{m}\;({}^{\circ}C)$	$2^{\mathrm{nd}}\;T_{m}\;({}^{\circ}C)$	$T_{c}\;({}^{\circ}C)$	$\Delta H_{c}\;(J/g)$	$T_{m}\;({}^{\circ}C)$	$T_{c}\;({}^{\circ}C)$	$\Delta H_{c}\;(J/g)$
1	51.6	87.6	69.1	56.8	85.5	69.1	57.1
3	50.8	86.4	68.8	52.2	85.6	68.9	49.7
5	51.4	87.1	69.0	40.0	84.4	69.0	40.5

**Table 2:** Flow activation energy based on Arrhenius equation.

Apparent Shear Rate (1/s)	MWCNTs (wt.%)	Activation Energy (k J/mol)
100	1	22.577
	3	19.123
	5	17.798
500	1	17.055
	3	15.325
	5	14.407
1000	1	14.534
	3	13.334
	5	12.558

**Table 3:** Tait parameters for EVA/MWCNT composite.

Parameter	Unit	1 wt.%	3 wt.%	5 wt.%
$b_{1m}$	mm^3^/g	1.1319 × 10^3^	1.1087 × 10^3^	1.0991 × 10^3^
$b_{2m}$	mm^3^/g·°C	8.0600 × 10^−1^	8.7920 × 10^−1^	8.2872 × 10^−^^1^
$b_{3m}$	bar	9.3649 × 10^2^	9.9568 × 10^2^	9.9742 × 10^2^
$b_{4m}$	1/°C	4.0226 × 10^-3^	4.3584 × 10^−3^	4.1905 × 10^−3^
$b_{5}$	°C	92.35	87.45	87.745
$b_{6}$	°C/bar	1.5788 × 10^−2^	1.9015 × 10^−2^	1.8815 × 10^−2^
$b_{1s}$	mm^3^/g	1.1037 × 10^3^	1.0889 × 10^3^	1.0789 × 10^3^
$b_{2s}$	mm^3^/g·°C	1.1518	1.1774	1.1390
$b_{3s}$	bar	8.3548 × 10^2^	9.8815 × 10^2^	9.8815 × 10^2^
$b_{4s}$	1/°C	1.1938 × 10^−2^	8.7264 × 10^−3^	8.7264 × 10^−3^
$b_{7}$	mm^3^/g	2.8788 × 10^1^	1.5930 × 10^1^	1.7689 × 10^1^
$b_{8}$	1/°C	3.6442 × 10^−2^	6.1900 × 10^−2^	5.5434 × 10^−2^
$b_{9}$	1/bar	3.3486 × 10^−4^	1.0705 × 10^−4^	9.2615 × 10^−4^

**Table 4:** Predicted elastic modulus and density for EVA/MWCNT composite.

MWCNTs (wt.%)	Young Modulus (MPa)		Density (g/cm^3^)	
	Halpin-Tsai Equation (S13)	Experiment	Rule-of-Mixture	Experiment
1	48.91	50.26 ± 1.64	0.94	0.932 ± 0.01
3	67.05	66.17 ± 1.38	0.95	0.942 ± 0.01
5	85.61	85.87 ± 3.07	0.96	0.952 ± 0.01

## Supplementary Materials

The following are available online at https://www.mdpi.com/2073-4360/11/8/1300/s1, Table S1. Injection molding L_9_ plan, Table S2. Parameters for resistivity measurements, Table S3. ANOVA for elastic modulus of EVA/MWCNT composite, Table S4. ANOVA for tensile strength of EVA/MWCNT composite, Table S5. ANOVA for stress at break of EVA/MWCNT composite, Table S6. ANOVA for strain at break of EVA/MWCNT composite, Table S7. ANOVA for electrical conductivity of EVA/MWCNT composite, Figure S1. Electrical resistivity measurement set-up. (a) Block diagram for electrical resistance measurement. (b) Data acquisition system.
