# Supplementary material for: Mechanical, Electrical and Rheological Behavior of Ethylene-Vinyl Acetate/Multi-Walled Carbon Nanotube Composites
Source: Polymers (Basel). 2019 Aug 2;11(8):1300. doi: 10.3390/polym11081300 (PMC6723935; doi:10.3390/polym11081300)
Supplement: Supplementary file 1 [file polymers-11-01300-s001.pdf]

polymers

Supplementary Materials

Mechanical, electrical and rheological behavior of ethylene-vinyl acetate/multi-walled carbon nanotube composites

Nicoleta-Violeta Stanciu, Felicia Stan*, Ionut-Laurentiu Sandu, Florin Susac, Catalin Fetecau, Razvan-Tudor Rosculet

Center of Excellence Polymer Processing, Dunarea de Jos University of Galati, 47 Domneasca, Galati, 800008, Romania

* Correspondence: felicia.stan@ugal.ro

1. Methods

1.1. Volume resistivity

The volume resistivity of the sample is calculated based on the geometric consideration from the following equation:

$$\rho = \frac{V}{I}\frac{A}{L} = R\frac{A}{L}, \tag{S1}$$

in which A is the cross-section area of the sample, L is the distance between the electrodes, and R is the measured resistance.

The resistance of the sample R is calculated by

$$R = \frac{R_0 \cdot R_m}{(R_0 - R_m)}, \tag{S2}$$

where the equivalent resistance of the circuit without sample R_0 is given by

$$R_0 = \frac{(V_m - V_{off})}{I}, \tag{S3}$$

and the measured resistance of the circuit with sample R_m (Ω) is given by

$$R_m = \frac{(V_m - V_{off})}{I}, \tag{S4}$$

in which V_m is the measured voltage (V), V_{off} is the measured voltage offset (V), and I is the source current (A).

2. Modeling the properties of EVA/MWCNT composites

2.1. pvT data

In general, the *pvT* behavior of polymers and polymer-based composites is described by the modified Tait equation [1–3]:

$$v(T,p) = v_0(T)\cdot\left[1 - C\cdot\ln\left(1+\frac{p}{B(T)}\right)\right] + v_t(T,p), \tag{S5}$$

where v_0 is the specific volume at zero pressure, v_t is the specific volume corresponding to crystalline phase, B is the sensitivity to pressure of polymer.

In the molten state, i.e., above the liquid-solid transition temperature, the following equations hold [1–4]:

$$v_0 = b_{1m} + b_{2m} \cdot (T - b_5), \tag{S6}$$

$$B(T) = b_{3m} \cdot \exp[-b_{4m}(T - b_5)], \tag{S7}$$

$$v_t(T, p) = 0 . \tag{S8}$$

In solid state, i.e., below the liquid-solid transition temperature, the polymer behavior is described by the following equations [1–4]:

$$v_0 = b_{1s} + b_{2s} \cdot (T - b_5), \tag{S9}$$

$$B(T) = b_{3s} \cdot \exp[-b_{4s}(T - b_5)], \tag{S10}$$

$$v_t(T, p) = b_7 \cdot \exp[b_8 \cdot (T - b_5) - b_9 \cdot p]. \tag{S11}$$

The parameters b_1 to b_4 describe the dependence on pressure and temperature in the melt and solid state. The b_5 and b_6 are parameters that describe the change of transition temperature with pressure, whereas b_7 to b_9 are parameters of semi-crystalline polymers that describe the form of the state transitions.

The liquid-solid transition temperature, which is the glass transition temperature for amorphous polymers and the melting or crystallization temperature for semi-crystalline polymers, can be described by [1–4]

$$T_t(p) = b_5 + b_6 \cdot p . \tag{S12}$$

The experimental data were divided into two states: melt and solid, and the parameters ($b_{1m} - b_{4m}$) and ($b_{1s} - b_{4s}$) were estimated from data corresponding to the respective states. Parameters b_5 and b_6 were estimated from data of transition temperature at different pressures.

2.2. Elastic modulus

To predict the Young's modulus of the EVA/MWCNT composite, the Halpin-Tsai model for randomly oriented nano-particles is applied [5–8]. According to this model, the Young's modulus of the composite can be predicted using the following equation [6,8]:

$$\frac{E_{EVA/MWCNT}}{E_{EVA}} = \frac{3}{8}\left[\frac{1+\varsigma\eta_L V_{MWCNT}}{1-\eta_L V_{MWCNT}}\right] + \frac{5}{8}\left[\frac{1+2\eta_T V_{MWCNT}}{1-\eta_T V_{MWCNT}}\right] \tag{S13}$$

with

$$\eta_L = \frac{E_{MWCNT}/E_{EVA} - 1}{E_{MWCNT}/E_{EVA} + \varsigma} \quad \text{and} \quad \eta_T = \frac{E_{MWCNT}/E_{EVA} - 1}{E_{MWCNT}/E_{EVA} + 2}, \tag{S14}$$

in which the exponential shape factor is given by [4,8,9]

$$\varsigma = \frac{2l}{d} e^{-a\, V_{MWCNT} - b}, \tag{S15}$$

where $E_{EVA/MWCNT}$ is the Young's modulus of the composite, E_{EVA} is the Young's modulus of the polymer matrix, E_{MWCNT} is the Young's modulus of the carbon nanotubes, d is the outside average diameter of the carbon nanotube, l is the length of the carbon nanotube, V_{MWCNT} is the volume fraction, and *a* and *b* are constants related to the degree of nanotube aggregation and account for the nonlinear behavior of the Halpin-Tsai equation [5,6].

2.3. Electrical conductivity

The power-law model is generally used to predict the electrical conductivity of polymer/CNT composites [10–14]. This model describes the dependence of the composite conductivity on the conductive filler above the percolation concentration.

Based on the assumption that the spatial distribution of nanotubes in the composite is uniform, the electrical conductivity can be written as [13]

$$\sigma_{DC} = \sigma_{MWCNT}\left(\varphi_{MWCNT} - \varphi_0\right)^t \text{ for } \varphi_{MWCNT} > \varphi_0, \quad \text{(S16)}$$

where σ_{DC} is the conductivity of the composite, σ_{MWCNT} is a parameter related to the conductivity of nanotubes, φ_{MWCNT} is the mass fraction of the nanotubes, φ_0 is the percolation threshold, i.e. the minimum quantity of the nanotubes to form a continuous network, and t is the critical exponent governing the dimensionality of the composites [15–18].

3. Tables

Table S1. Injection molding L_9 plan.

Experiment	Melt temperature (°C)	Injection pressure (MPa)
1		70
2	140	80
3		90
4		70
5	160	80
6		90
7		70
8	180	80
9		90

Table S2. Parameters for resistivity measurements.

Injection pressure (MPa)	1 wt.% (nA)	3 wt.% (μA)	5 wt.% (μA)
	0.0002	0.06	20.0
70	0.0010	0.20	100.0
	0.0020	0.60	200.0
	0.0002	3.0	20.0
90	0.0010	10.0	100.0
	0.0020	30.0	200.0

Table S3. ANOVA for elastic modulus of EVA/MWCNT composite.

Source	DF	Seq SS	Adj SS	Adj MS	F	P
MWCNTs (wt.%)	2	17511.4	17511.4	8755.72	1697.28	0
Crosshead speed (mm/min)	2	935.4	935.4	467.68	90.66	0
Melt temperature (°C)	2	332.7	332.7	166.36	32.25	0
MWCNTs × Melt temperature	4	222.2	222.2	55.54	10.77	0
Crosshead speed × Melt temperature	4	71	71	17.76	3.44	0.013
**Residual Error**	**66**	**340.5**	**340.5**	**5.16**		
**Total**	**80**	**19413.2**				

Table S4. ANOVA for tensile strength of EVA/MWCNT composite.

Source	DF	Seq SS	Adj SS	Adj MS	F	P
MWCNTs (wt.%)	2	36.6538	36.6538	18.3269	257.79	0
Crosshead speed (mm/min)	2	7.1316	7.1316	3.5658	50.16	0
Melt temperature (°C)	2	0.2829	0.2829	0.1415	1.99	0.145
MWCNTs × Crosshead speed	4	1.072	1.072	0.268	3.77	0.008
MWCNTs ×Melt temperature	4	6.1001	6.1001	1.525	21.45	0
Crosshead speed × Melt temperature	4	3.2698	3.2698	0.8174	11.5	0
**Residual Error**	**62**	**4.4077**	**4.4077**	**0.0711**		
**Total**	**80**	**58.9179**				

Table S5. ANOVA for stress at break of EVA/MWCNT composite.

Source	DF	Seq SS	Adj SS	Adj MS	F	P
MWCNTs (wt.%)	2	20.471	20.471	10.2355	166.74	0
Crosshead speed (mm/min)	2	13.83	13.83	6.9151	112.65	0
Melt temperature (°C)	2	2.656	2.656	1.3279	21.63	0
MWCNTs × Crosshead speed	4	1.356	1.356	0.3391	5.52	0.001
MWCNTs ×Melt temperature	4	6.059	6.059	1.5148	24.68	0
Crosshead speed × Melt temperature	4	1.716	1.716	0.4291	6.99	0
**Residual Error**	**62**	**3.806**	**3.806**	**0.0614**		
**Total**	**80**	**49.895**				

Table S6. ANOVA for strain at break of EVA/MWCNT composite.

Source	DF	Seq SS	Adj SS	Adj MS	F	P
MWCNTs (wt.%)	2	25.8057	25.8057	12.9029	1404.48	0
Crosshead speed (mm/min)	2	0.4932	0.4932	0.2466	26.84	0
Melt temperature (°C)	2	8.507	8.507	4.2535	463	0
MWCNTs × Crosshead speed	4	0.1689	0.1689	0.0422	4.6	0.003
MWCNTs ×Melt temperature	4	0.569	0.569	0.1422	15.48	0
Crosshead speed × Melt temperature	4	0.1267	0.1267	0.0317	3.45	0.013
**Residual Error**	**62**	**0.5696**	**0.5696**	**0.0092**		
**Total**	**80**	**36.2401**				

Table S7. ANOVA for electrical conductivity of EVA/MWCNT composite.

Source	DF	Seq SS	Adj SS	Adj MS	F	P
MWCNTs (wt.%)	2	8.38E-03	8.38E-03	4.19E-03	178.53	0
Melt temperature (°C)	2	3.96E-04	3.96E-04	1.98E-04	8.43	0.037
Injection pressure (MPa)	1	3.30E-05	3.30E-05	3.30E-05	1.41	0.301
MWCNTs×Melt temperature	4	7.09E-04	7.09E-04	1.77E-04	7.55	0.038
MWCNTs × Injection pressure	2	1.70E-05	1.70E-05	9.00E-06	0.36	0.717
Melt temperature × Injection pressure	2	3.00E-05	3.00E-05	1.50E-05	0.65	0.57
**Residual Error**	**4**	**9.40E-05**	**9.40E-05**	**2.30E-05**		
**Total**	**17**	**9.66E-03**				

4. Figures

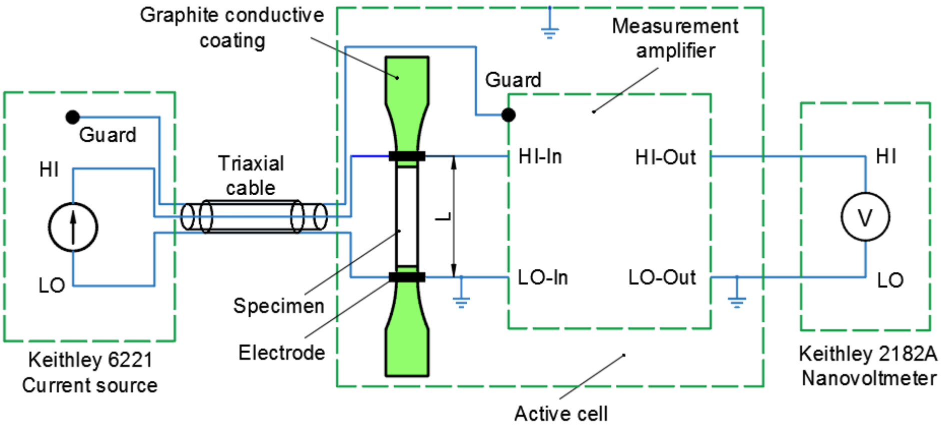

(a)

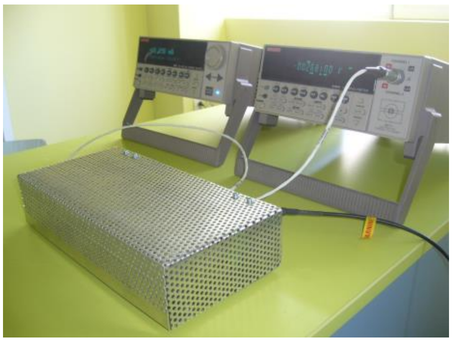

(b)

Figure S1. Electrical resistivity measurement set-up. (a) Block diagram for electrical resistance measurement. (b) Data acquisition system.

References

1. Mekhilef, N. Viscoelastic and pressure–volume–temperature properties of poly(vinylidene fluoride) and poly(vinylidene fluoride)–hexafluoropropylene copolymers. *J. Appl. Polym. Sci.* **2001**, *80*, 230–241.
2. Chang, R.Y.; Chen, C.H.; Su, K.S. Modifying the Tait equation with cooling-rate effects to predict the pressure– volume–temperature behaviors of amorphous polymers: Modeling and experiments. *Polym. Eng. Sci*. **1996**, *36*, 1789-1795.
3. Utracki, L.A. PVT of amorphous and crystalline polymers and their nanocomposites. *Polym. Degrad. Stabil.* **2010**, *95*, 411–421.
4. Rodgers, P.A. Pressure–volume–temperature relationships for polymeric liquids: A review of equations of state and their characteristic parameters for 56 polymers. *J. Appl. Polym. Sci.* **1993**, *48*, 1061–1080.
5. Yeh, M.-K.; Tai, N.-H.; Liu, J.-H. Mechanical behavior of phenolic-based composites reinforced with multi-walled carbon nanotubes. *Carbon* **2006**, *44*, 1–9.
6. Stan, F.; Sandu, L.I.; Fetecau, C. Effect of processing parameters and strain rate on mechanical properties of carbon nanotube-filled polypropylene nanocomposites. *Compos. Part B Eng.* **2014**, *59*, 109–122.
7. Peeterbroeck, S.; Breugelmans, L.; Alexandre, M.; BNagy, J.; Viville, P.; Lazzaroni, R.; Dubois, P. The influence of the matrix polarity on the morphology and properties of ethylene vinyl acetate copolymers-carbon nanotube nanocomposites. *Compos. Sci. Technol.* **2007**, *67*, 1659–1665.
8. Halpin, J.C.; Kardos, J.L. The Halpin–Tsai equations: A review. *Polym. Eng. Sci.* **1976**, *16*, 344–352.
9. Stan, F.; Fetecau, C.; Stanciu, N.V.; Rosculet, R.T.; Sandu, L.I. Investigation of structure-property relationships in thermoplastic polyurethane/multiwalled carbon nanotube composites. In Proceedings of the ASME 2017 12th International Manufacturing Science and Engineering Conference, 2, V002T03A016, Los Angeles, CA, USA, 4–8 June 2017, doi:10.1115/MSEC2017-2760.

10. Vargas-Bernal, R.; Herrera-Pérez, G.; Calixto-Olalde, E.; Tecpoyotl-Torres, M. Analysis of DC electrical conductivity models of carbon nanotube-polymer composites with potential application to nanometric electronic devices. *J. Electr. Comput. Eng.* **2013**, *2013*, 179538.
11. Taherian, R. Development of an equation to model electrical conductivity of polymer-based carbon nanocomposites. *ECS J. Solid State Sci. Technol.* **2014**, *3*, M26–M38.
12. Musumeci, A.W.; Silva, G.G.; Liu, J.-W.; Martens, W.N.; Waclawik, E.R. Structure and conductivity of multi-walled carbon nanotube/poly(3-hexylthiophene) composite films. *Polymer* **2007**, *48*, 1667–1678.
13. Stauffer, D; Aharony, A. *Introduction to the Percolation Theory*, 2nd ed.; Francis and Taylor: London, UK, 1991; pp. 1–14, 73–86.
14. Bao, W.S.; Meguid, S.A.; Zhu, Z.H.; Meguid, M.J. Modeling electrical conductivities of nanocomposites with aligned carbon nanotubes. *Nanotechnology* **2011**, *22*, 485704.
15. Grimaldi, C.; Balberg, I. Tunneling and nonuniversality in continuum percolation systems. *Phys. Rev. Lett.* **2006**, *96*, 066602.
16. Baskin, E. Universal critical exponents in percolation systems with tunneling. *J. Non Cryst. Solids* **2006**, *352*, 1117–1121.
17. Zhang, R.; Baxendale, M.; Peijs, T. Universal resistivity-strain dependence of carbon nanotube/polymer composites. *Phys. Rev. B* **2007**, *76*, 195433.
18. Kim, Y.J.; Shin, T.S.; Choi, H.D.; Kwon, J.H.; Chung, Y.-C.; Yoon, H.G. Electrical conductivity of chemically modified multiwalled carbon nanotube/epoxy composites. *Carbon* **2005**, *43*, 23–30.
